# Cerebrospinal fluid (CSF) biomarkers of iron status are associated with CSF viral load, antiretroviral therapy, and demographic factors in HIV-infected adults

**DOI:** 10.1186/s12987-017-0058-1

**Published:** 2017-04-21

**Authors:** Stephanie M. Patton, Quan Wang, Todd Hulgan, James R. Connor, Peilin Jia, Zhongming Zhao, Scott L. Letendre, Ronald J. Ellis, William S. Bush, David C. Samuels, Donald R. Franklin, Harpreet Kaur, Jennifer Iudicello, Igor Grant, Asha R. Kallianpur

**Affiliations:** 1 Department of Neurosurgery, Penn State Hershey Medical Center, 500 University Drive, Mailbox H110, Hershey, PA 17033 USA; 2 Department of Biomedical Informatics, Vanderbilt University School of Medicine, Nashville, TN USA; 3 Department of Medicine, Vanderbilt University School of Medicine, Nashville, TN USA; 4 School of Biomedical Informatics, The University of Texas Health Science Center at Houston, Houston, TX USA; 5 Department of Medicine, University of California-San Diego, San Diego, CA USA; 6 Department of Neurology, University of California-San Diego, San Diego, CA USA; 7 Department of Epidemiology and Biostatistics, Case Western Reserve University School of Medicine, Cleveland, OH USA; 8 Department of Molecular Physiology and Biophysics, Vanderbilt University, Nashville, TN USA; 9 Genomic Medicine Institute, Cleveland Clinic/Lerner Research Institute, Cleveland, OH USA; 10 Department of Psychiatry, University of California-San Diego, San Diego, CA USA; 11 Department of Molecular Medicine, Cleveland Clinic Lerner College of Medicine of Case Western Reserve University, Cleveland, OH USA

**Keywords:** Iron transport, Cerebrospinal fluid, HIV, Transferrin, H-ferritin, HIV-associated neurocognitive disorders

## Abstract

**Background:**

HIV-associated neurocognitive disorder (HAND) remains common, despite antiretroviral therapy (ART). HIV dysregulates iron metabolism, but cerebrospinal fluid (CSF) levels of iron and iron-transport proteins in HIV-infected (HIV+) persons are largely unknown. The objectives of this study were to characterize CSF iron-related biomarkers in HIV+ adults and explore their relationships to known predictors of HAND.

**Methods:**

We quantified total iron, transferrin and heavy-chain (H)-ferritin by immunoassay in CSF sampled by lumbar puncture in 403 HIV+ participants in a multi-center, observational study and evaluated biomarker associations with demographic and HIV-related correlates of HAND [e.g., age, sex, self-reported race/ethnicity, ART, and detectable plasma virus and CSF viral load (VL)] by multivariable regression. In a subset (N = 110) with existing CSF: serum albumin (Q_Alb_) measurements, Q_Alb_ and comorbidity severity were also included as covariates to account for variability in the blood–CSF-barrier.

**Results:**

Among 403 individuals (median age 43 years, 19% women, 56% non-Whites, median nadir CD4+ T cell count 180 cells/µL, 46% with undetectable plasma virus), men had 25% higher CSF transferrin (median 18.1 vs. 14.5 µg/mL), and 71% higher H-ferritin (median 2.9 vs. 1.7 ng/mL) than women (both *p*-values ≤0.01). CSF iron was 41% higher in self-reported Hispanics and 27% higher in (non-Hispanic) Whites than in (non-Hispanic) Blacks (median 5.2 and 4.7 µg/dL in Hispanics and Whites, respectively, vs. 3.7 µg/dL in Blacks, both *p* ≤ 0.01); these findings persisted after adjustment for age, sex, and HIV-specific factors. Median H-ferritin was 25% higher (*p* < 0.05), and transferrin 14% higher (*p* = 0.06), in Whites than Blacks. Transferrin and H-ferritin were 33 and 50% higher, respectively, in older (age > 50 years) than in younger persons (age ≤ 35 years; both *p* < 0.01), but these findings lost statistical significance in subset analyses that adjusted for Q_Alb_ and comorbidity. After these additional adjustments, associations were observed for CSF iron and transferrin with race/ethnicity as well as CSF VL, for transferrin with sex and ART, and for H-ferritin with plasma virus detectability and significant comorbidity (all *p* < 0.05).

**Conclusions:**

CSF iron biomarkers are associated with demographic factors, ART, and CSF VL in HIV+ adults. Future studies should investigate a role for CNS iron dysregulation, to which an altered blood-CSF barrier may contribute, in HAND.

**Electronic supplementary material:**

The online version of this article (doi:10.1186/s12987-017-0058-1) contains supplementary material, which is available to authorized users.

## Background

HIV affects an estimated 40 million people worldwide [1], and owing to the success of combination antiretroviral therapy (ART) in suppressing viral replication, this population is aging [2–4]. An increasing number of chronic disease complications, such as HIV-associated neurocognitive disorders (HAND), are therefore the focus of current efforts to improve quality of life for HIV-infected (HIV+) individuals. HAND remains disturbingly common despite ART, for reasons that are only partially understood, occurring in 30–60% of unselected HIV+ persons, even when the virus is undetectable [5, 6]. Intensive research has suggested many possible mechanisms and etiologies for HAND, including persistent low-level immune activation and inflammation within the central nervous system (CNS) [4, 7, 8], comorbid conditions [9], and accelerated aging [10], but few of these mechanisms are directly actionable, and effective interventions remain elusive. Risk factors for HAND include the nadir CD4+ T-cell count, indicating the depth of immunosuppression achieved during the course of HIV disease, older age at seroconversion, anemia, delay in initiating ART, and the presence of HIV RNA in the CSF and plasma, as well as comorbidity [11–14]. Anemia is prevalent in HIV infection, even when the virus is undetectable during successful ART, and it has been consistently associated with increased morbidity and mortality in HIV infection in retrospective and prospective studies [15]. In addition, the possibility that CNS toxicity due to ART contributes to HAND remains a matter of debate; certain antiretroviral drugs such as zidovudine (ZDV) have well-described effects on iron transport and frequently cause anemia, and others may exhibit neurotoxicity [14, 16, 17]. Female sex appears also to be associated with higher susceptibility to HAND, but women have been underrepresented in prior HIV studies, and factors unrelated to HIV infection may also explain this finding [18–20]. Finally, HIV+ persons of Hispanic ethnicity experience an increased risk of neurocognitive decline, possibly due in part to reduced or delayed access to health care [13, 18, 21–23].

Iron homeostasis is essential for normal brain function: iron is required for the synthesis of monoamine neurotransmitters, for mitochondrial function, myelin synthesis, and the catalytic function of a large number of metabolic enzymes [4, 24]. Alterations in brain iron content and distribution (e.g., excess iron deposition) have long been associated with complex neurodegenerative disorders such as Parkinson’s disease, Alzheimer’s disease, and amyotrophic lateral sclerosis (ALS) [25]. Neurocognitive impairment has also been reported in the restless leg syndrome (RLS), in which brain iron deficiency contributes to dopaminergic dysfunction within the brain [26]. It has been challenging to determine the extent to which iron plays a pathogenic as opposed to a bystander role in these conditions, but evidence is accumulating in favor of the former [27–30]. An extensive literature has established that peripheral iron deficiency also leads to reduced cognitive performance and behavioral disturbances, which in children and adolescents may be long-lasting and not fully reversible upon iron repletion [31–33]. Despite the known importance of iron to brain health, iron and iron transport proteins in the CSF have been measured only anecdotally in small numbers of HIV-negative persons with specific neurodegenerative conditions, or in healthy controls [34–38]. CNS iron homeostasis has not been adequately investigated in HIV+ individuals with or without HAND, either by measurement of iron or iron-related biomarkers in CSF, or indirectly, by iron-sensitive neuroimaging [4, 39, 40].

Transferrin, the chief iron-transporter in the circulation, binds and transports ferric iron in a soluble, nonreactive state to metabolically active cells throughout the body, whereupon transferrin-bound iron is internalized by receptor-mediated endocytosis [41]. Transferrin is synthesized and secreted by the choroid plexus and is also produced by oligodendrocytes; it is the chief mode of delivery of iron to neurons [42]. Ferritin, the principal intracellular iron-storage molecule in tissues, is frequently elevated in HIV+ persons, possibly reflecting a prolonged acute-phase inflammatory response [43, 44]. Ferritin comprises varying proportions of heavy (iron-avid) and light (relatively iron-poor) chains (H- and L-ferritin, respectively), depending on the tissue of origin. H-ferritin is the predominant form in the CNS, and it delivers iron to oligodendrocytes via the Tim-2 receptor [45, 46]. Iron delivery by H-ferritin to other cell types has also been suggested [47]. Serum ferritin levels generally rise, and transferrin levels fall, with increasing systemic iron stores; the reverse changes occur in functionally iron-deficient states [41, 43]. Both transferrin and H-ferritin can be influenced by inflammatory stimuli, such as infection or comorbid conditions [48, 49]. The synthesis, transport, and regulation of iron, transferrin, and ferritin within the CNS are incompletely understood and the subject of ongoing investigation [24] In contrast to many other serum proteins, levels of these iron-related proteins in CSF have often been reported not to correlate with those in the peripheral circulation, and the brain is well buffered against systemic iron deficiency or excess [50]. Although a number of studies have examined peripheral (serum or plasma) iron biomarkers in HIV infection as a factor in disease outcomes [51–54], CSF levels of iron, transferrin, and H-ferritin remain inadequately characterized from an epidemiological perspective [55, 56].

Serum is the most convenient source for routine, noninvasive testing of iron parameters, but the CSF is more likely to provide an accurate reflection of iron transport in the CNS. There is also considerable precedent for the use of CSF biomarkers as surrogate markers of iron metabolism and/or neuronal injury in the brain [57–59]. Recent studies have documented selective prioritization and partitioning of iron across different tissues in the anemic state, for example, and thus, blood indices may not reflect CNS iron depletion [60, 61]. Another advantage of CSF as the biological medium for estimating CNS iron status is that CSF is sequestered behind both the blood–brain barrier (BBB) and blood–CSF barrier. This isolation from the periphery permits measurement of iron and iron-management proteins at least in part derived from or in equilibrium with the brain parenchyma and specific to the CNS, potentially reflecting unique aspects of iron transport within that compartment.

It is of interest to evaluate associations of known predictors of neurocognitive decline in HIV infection with levels of CSF iron and iron-transport proteins, because such associations would suggest a possible novel role for dysregulation of iron metabolism or iron transport pathways in the pathogenesis of HAND that might be targeted therapeutically. Analogous approaches have proven instructive in achieving a better understanding of other chronic disease phenotypes linked to inflammation, such as cancer [62]. The purpose of this study was therefore to quantify, for the first time, iron and biomarkers of iron transport in the CSF of HIV+ persons and systematically evaluate their relationships to host factors such as age, sex, ethnicity or ancestry, and key HIV disease-related factors like ART (including ZDV use) and the presence and quantity of CSF virus.

## Methods

### Patient selection and CSF sampling

The CNS HIV Antiretroviral Therapy Effects Research (CHARTER) Study is an observational, prospective cohort study that enrolled ambulatory, HIV+ adults at six large U.S. Medical Centers in the U.S., including Johns Hopkins University School of Medicine (Baltimore, MD, USA), the University of California (San Diego, CA, USA), University of Texas Medical Branch (Galveston, TX, USA), Washington University School of Medicine (Saint Louis, MO, USA), University of Washington (Seattle, WA, USA), and the Icahn School of Medicine of Mount Sinai (New York, NY, USA). CHARTER was designed specifically to evaluate neurological outcomes in HIV+ individuals; hence, all participants underwent detailed neuromedical, neuropsychological, and cognitive evaluations at baseline and 6-month follow-up visits, as previously described [7]. Comorbid conditions were categorized upon careful assessment as minimal, mild or moderate, or severe by expert clinicians [6, 63].

Participants in this study of CSF iron biomarkers were selected based on availability of at least 1.0 mL of CSF at both baseline (N = 403) and 6-month (N = 100) follow-up visits. Individuals with comorbid conditions deemed by expert clinicians to be severe (conditions such as traumatic brain injury with prolonged loss of consciousness, recent stroke, or a history of developmental delay) were excluded, and the remainder were classified as having minimal or mild-moderate comorbidity following extensive chart review [6]. Basic demographic information (e.g., age, sex, self-reported race/ethnicity) and HIV disease characteristics [e.g., ART, nadir CD4+ T-cell count, and HIV RNA concentrations (viral load, VL) in plasma and CSF] were determined at the baseline visit during structured in-person interviews, when CSF was first sampled, as described in detail previously [13]. Non-Hispanic Whites of European ancestry are henceforth referred to as “Whites” and non-Hispanic Blacks, as “Blacks”, to distinguish them from self-reported Hispanic individuals. Nine study participants reported their race/ethnicity as “Other” and were too few in number for subgroup analyses; therefore, race-stratified analyses included only self-reported Black, White, and Hispanic study participants.

All CHARTER study participants provided written informed consent for the study, and the CHARTER study adheres to the ethical principles set forth in the Declaration of Helsinki. The present study was also approved by the institutional review boards of all participating institutions.

### CSF iron quantification

Iron content within the CSF was evaluated using the Quantichrom iron assay (BioAssay Systems, Hayward, CA, USA) and the manufacturer’s protocol. Briefly, 50 μL of standards or patient CSF samples were mixed with 200 μL Quantichrom Working Reagent in a 96-well plate (in duplicate) and incubated at room temperature for 40 min. The iron concentration in experimental samples was determined by comparison of the optical density at 590 nm with the standard curve.

### CSF transferrin quantification

Transferrin content within sampled CSF was determined by enzyme-linked immunosorbent assay (Human Transferrin ELISA kit (ab108902) (Abcam, Cambridge, MA, USA) performed according to the manufacturer’s protocol. After the CSF was collected and centrifuged, the samples (in duplicate) were diluted 1:1000 with mix diluent. Briefly, transferrin standard or diluted CSF sample (50 μL) was added to each well and the solution was incubated for 2 h. Following incubation, the plate was washed five times with 200 μL of 1× Wash Buffer. Following the wash step, 1× Biotinylated transferrin antibody (50 μL) was added to each well and the solution was incubated for 1 h. The plate was washed again as described above. 1× SP Conjugate (50 μL) was added to each well and the solution was incubated for 30 min. The microplate was washed again as described above. Chromogen substrate (50 μL) was added to each well and the solution was incubated for 12 min. Stop solution (50 μL) was added to each well and the absorbance was read immediately on a microplate reader at 450 nm. Transferrin concentration was then determined by comparison to the standard curve.

### CSF H-ferritin quantification

The H-ferritin content of CSF was determined by an enzyme-linked immunosorbent assay (Human H-ferritin ELISA kit, Abnova, Taipei, Taiwan) performed according to the supplier’s instructions. Briefly, 20 µL of standards, subject samples (in duplicate), and controls were combined with 100 µL of enzyme conjugate reagent. The solution was mixed for 30 s and incubated at room temperature for 45 min. The incubation mixture was removed and the plate was rinsed five times with distilled water. The TMB Reagent (100 µL) was added into each well, the solution was mixed for 10 s, and the solution was incubated at room temperature in the dark for 20 min. Stop Solution (100 µL) was added to each well and the solution was mixed for 30 s. The ferritin concentration in the experimental samples was determined by comparison of the optical density at 450 nm with the standard curve within 15 min.

### Serum iron biomarker measurement

Iron, ferritin, and transferrin were also quantified in serum using routine, commercially available, automated assays in a subset of study participants (N = 11) who had sufficient serum available at the 6-month follow-up visit. Circulating ferritin, which consists primarily of the L subunit, was quantified by electrochemiluminescence assay (“ECLIA”), using a biotinylated, monoclonal ferritin-specific antibody and a ruthenium-labeled monoclonal ferritin antibody to form a sandwich complex. Upon addition of streptavidin-coated microparticles, chemiluminescence emission was measured by a photomultiplier (Roche e411 immunoassay analyzer, Roche Diagnostics, Indianapolis, IN, USA) and ferritin concentration determined from a standard curve. Serum iron and transferrin were measured on a Roche c311 automated chemistry analyzer (Roche Diagnostics, Indianapolis, IN, USA), iron by a colorimetric method based on the FerroZine reaction (without deproteinization), which captures unbound and transferrin-bound iron, and transferrin by immunoturbidimetry [64].

### Functional integrity of the blood–CSF barrier

As a measure of the integrity of the blood–CSF barrier, CSF and matched serum levels of albumin were quantified by the nephelometric method (Dade Behring BNII, Deerfield, IL, USA) in CHARTER study subjects, 110 of whom participated in this study, and the ratio of CSF to serum albumin (CSF: serum albumin ratio, or Q_Alb_) was calculated as Q_Alb_ = albumin(CSF)/albumin(serum) [65]. Individuals with matched CSF and serum albumin measurements from multiple visits were assigned a mean value for Q_Alb_.

### Statistical analyses

The Wilcoxon rank sum test was used to determine univariate differences in CSF iron, H-ferritin, and transferrin levels among HIV-infected individuals by demographic and clinical characteristics or factors. Spearman’s *rho* values were determined for correlation between CSF iron biomarkers at baseline or at 6-months, and between CSF and corresponding serum biomarker levels at the 6-month visit in the subset with matched measurements. Unadjusted and adjusted analyses of association were performed after taking the natural logarithm of biomarker values to enforce normality, in order to fulfill the assumptions of linear regression.

Associations of each CSF biomarker with age, sex, and race/ethnicity (dichotomized as Blacks compared with Whites, Whites compared with Hispanics, or Blacks compared with Hispanics), and clinical HIV disease characteristics were individually evaluated in multivariable regression models. Clinical disease factors that were evaluated included: VL in plasma and CSF and presence or absence of detectable plasma or CSF virus, estimated duration of HIV infection (months), ART (on vs. off therapy at the time of blood sampling), current ZDV use (yes/no), nadir CD4+ T-cell count, AIDS status (yes/no), hemoglobin level, anemia status (yes/no), current alcohol use (yes/no), and comorbidity (minimal vs. mild-to-moderate). Hemoglobin, anemia status, and alcohol use showed no relationship to CSF iron biomarkers in univariate analyses and were therefore not included in models to optimize power. Regression models for iron, transferrin, and H-ferritin are presented with partial or full covariate adjustment. For CSF iron, models included the following covariates: (1) age, sex, and race/ethnicity (partial adjustment), or all of the covariates listed in (1) as well as ART (on vs. off), current ZDV use (yes vs. no), plasma VL detectability (yes vs. no), and CSF VL (full adjustment). Covariates in partially adjusted models of transferrin and H-ferritin included age, sex, race/ethnicity, and ZDV use; ART, plasma VL detectability and CSF VL were added to fully adjusted models. *Beta*-coefficients and their 95% Confidence Intervals were estimated for associations, and two-sided *p*-values were determined. Following analyses in the entire study sample of 403 individuals, similar analyses with additional adjustment for mean Q_Alb_ were performed in the 110 individuals who had this data. All analyses were performed using STATA statistical software v10.0.

## Results

### CSF iron-related biomarkers in CHARTER study participants

**Fig. 1:**
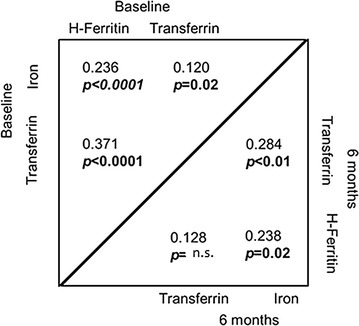
Spearman correlations between different CSF iron biomarkers in CHARTER study participants at baseline and at 6 months. *p*-values <0.05 (*bolded*) are considered statistically significant

**Fig. 2:**
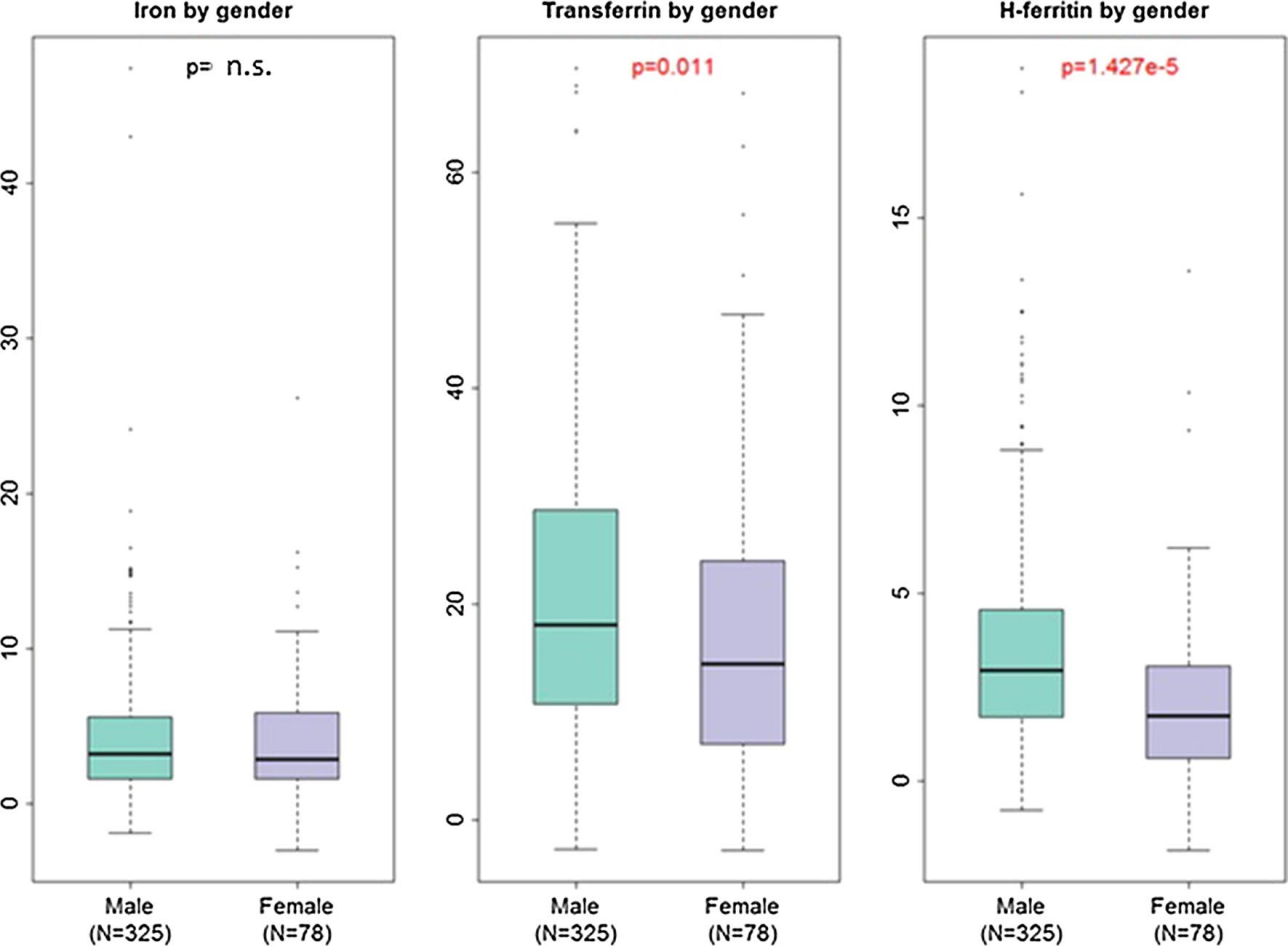
Boxplots of CSF iron biomarker distribution in CHARTER participants based on sex. *p*-values <0.05 are statistically significant and indicated in *red*. Units of measurement are as follows: iron (µg/dL), transferrin (µg/mL), H-ferritin (ng/mL)

Characteristics of the CHARTER study population at baseline are summarized in Table 1. Median CSF iron levels in this sample were 3.1 µg/dL (interquartile range or IQR 1.6–5.7 µg/dL); median transferrin levels were 17.2 µg/mL (IQR 10.3–27.6 µg/mL), and median H-ferritin levels were 2.7 ng/mL (IQR 1.4–4.2). In order to characterize demographic differences in CSF iron-related biomarkers in HIV+ persons, we measured CSF iron, transferrin, and H-ferritin in 403 cryopreserved CSF samples from CHARTER study participants collected at baseline and in 100 of the same persons at 6 months of follow-up. Within individuals, CSF iron, transferrin and ferritin levels measured at the baseline visit were correlated with levels of the same biomarkers measured at the 6-month visit (all *p* < 0.01, see Additional file 1: Figure S1 and Additional file 2: Table S1). Correlations between different iron-related biomarkers at baseline, and the same correlations at 6 months are shown in Fig. 1. CSF iron levels were weakly correlated with CSF transferrin and H-ferritin levels at baseline and 6 months (Spearman’s *rho* = 0.12 and 0.24 at baseline, and *rho* = 0.28 and 0.24 at 6 months, respectively, all *p*-values <0.05). Transferrin levels in CSF were modestly correlated with H-ferritin levels at baseline (*rho* = 0.37, *p* < 0.05), but not at 6 months. Age, sex, and ZDV use did not differ between study participants sampled at baseline and 6-months. As shown in Table 2 and Fig. 2, CSF transferrin and H-ferritin levels differed significantly between men and women in the study in univariate analyses (median transferrin levels in men at the baseline visit were 18.1 vs. 14.5 µg/mL, *p* < 0.05; median H-ferritin levels were 2.9 vs. 1.7 ng/mL, *p* = 1.4 × 10^−5^). Significant sex differences in CSF iron content were not observed.

**Table 1:** Summary of demographic and HIV disease characteristics of CHARTER study participants at baseline (entry) whose iron biomarkers were measured

Variable at baseline visit	All participants N = 403
Age (years), median (IQR)	43 (39, 49)
Sex (N, % women)	78 (19)
Race/ethnicity by self-report (N, %)	
Non-Hispanic Black	174 (43.2)
Non-Hispanic White	178 (44.2)
Hispanic	42 (10.4)
Other	9 (2.2)
Estimated duration of HIV infection (months), median (IQR)	118 (57, 183)
Nadir CD4+ T-cell count, median (IQR)	180 (56, 315)
AIDS status (N, %)	245 (61)
Virus detectability in plasma (N, % >LLQ)	215 (54)
Virus detectability in CSF (N, % >LLQ)	126 (31)^a^
CSF VL (log_10_ HIV RNA copies/mL), median (IQR)	1.7 (1.7, 2.2)^a^
ZDV use (N, % yes)	79 (20)
Comorbidity (% mild to moderate)	134 (33)
Current alcohol abuse (N, % yes)	10 (2.5)
Hemoglobin, mean ± SD	14.1 ± 1.5
Anemia (N, %)	54 (14)
CSF iron (µg/dL), median (IQR)^b^	3.1 (1.6, 5.7)
CSF transferrin (µg/mL), median (IQR)^b^	17.2 (10.3, 27.6)
CSF ferritin (ng/mL), median (IQR)^b^	2.7 (1.4, 4.2)

*IQR* interquartile range, *LLQ* lower limit of quantitation, *CSF* cerebrospinal fluid, *VL* viral load (HIV RNA), *ZDV* zidovudine, *SD* standard deviation, *µg/ng//dL/mL* micrograms or nanograms per deciliter or milliliter

^a^Three individuals did not have CSF viral load measurements

^b^Values of zero for iron biomarkers were at or below the lower limit of detection of the assay

**Table 2:** Distributions of iron biomarkers in CSF at the baseline visit by sex [median value (interquartile range, IQR)]

CSF biomarker (units)	Men (N = 325)	Women (N = 78)	*p* **-**value
Iron (µg/dL)	3.25 (1.60, 5.63)	2.84 (1.60, 5.88)	n.s.
Transferrin (µg/mL)	18.07 (10.80, 28.74)	14.47 (6.99, 24.03)	*0.01*
H-ferritin (ng/mL)	2.94 (1.69, 4.55)	1.72 (0.61, 3.05)	*<0.0001*

*µg/dL* micrograms per deciliter, *µg/mL* micrograms per milliliter, *ng/mL* nanograms per milliliter

*p*-values <0.05 (italics) are statistically significant

**Fig. 3:**
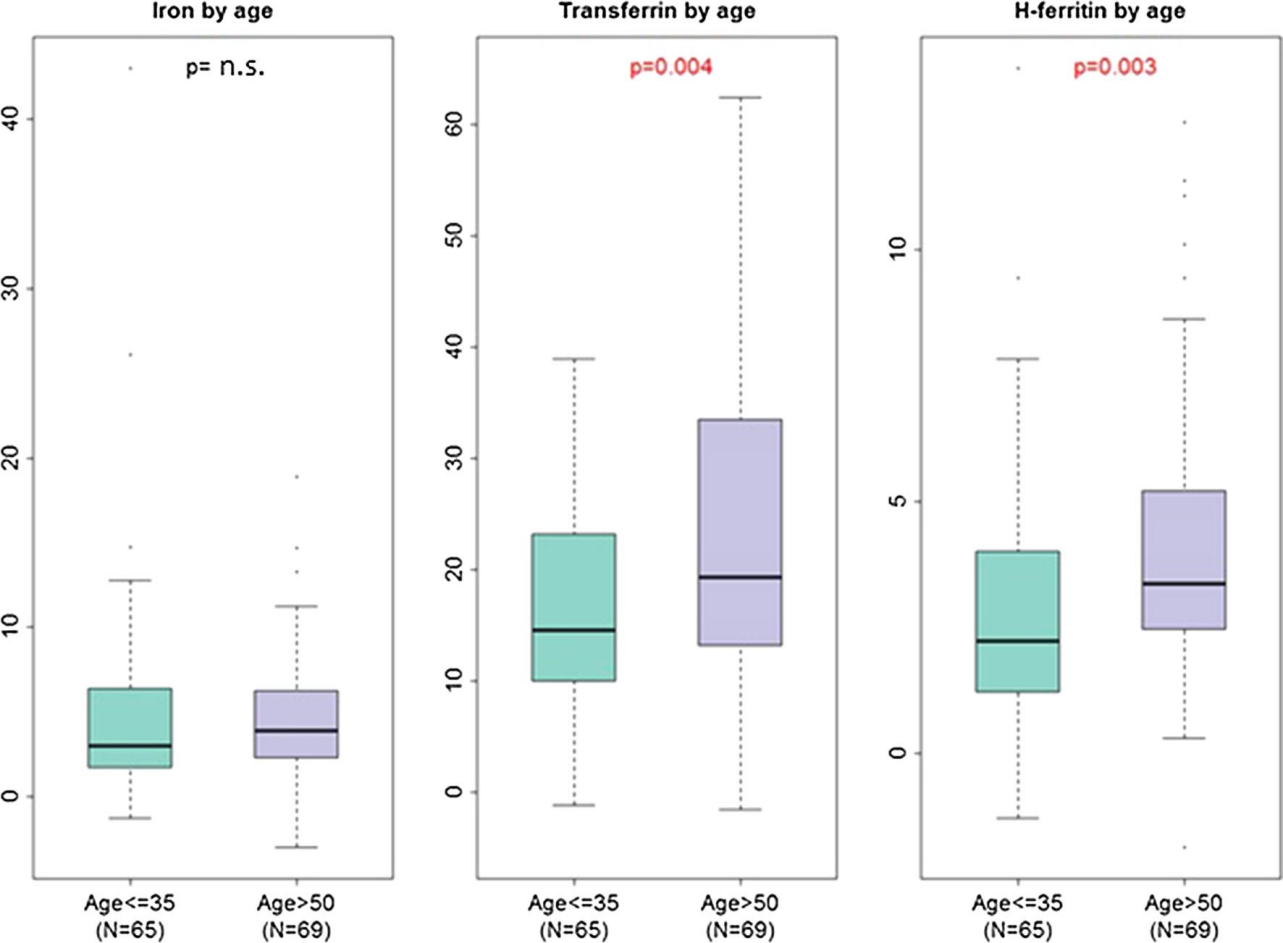
Boxplots of CSF iron biomarker distributions in CHARTER subjects 35 years or younger, or older than 50 years of age. *p*-values <0.05 are indicated in *red*. Units of measurement: iron (µg/dL), transferrin (µg/mL), H-ferritin (ng/mL)

In order to better explore age-related differences in CSF iron measures, we evaluated CSF iron, transferrin, and H-ferritin levels in non-contiguous age groups of younger (≤35 years) and older HIV+ adults (>50 years of age) from the CHARTER Study. As shown in Table 3 and Fig. 3, 134 study participants were included in this comparison, which was not adjusted for other HIV-related or demographic factors. CSF transferrin and H-ferritin levels were significantly higher in older than in younger individuals in this study population (both *p*-values <0.01), but CSF total iron content did not differ significantly between these groups.

**Table 3:** CSF iron biomarker distributions at baseline [median (interquartile range)] in younger as compared to older CHARTER subjects

CSF biomarker	Age ≤ 35 (N = 65)	Age > 50 (N = 69)	*p* **-**value
Iron (µg/dL)	3.00 (1.75, 6.37)	3.88 (2.30, 6.25)	n.s.
Transferrin (µg/mL)	14.51 (9.96, 23.16)	19.29 (13.21, 33.42)	*<0.01*
H-ferritin (ng/mL)	2.24 (1.22, 4.01)	3.37 (2.47, 5.21)	*<0.01*

**Fig. 4:**
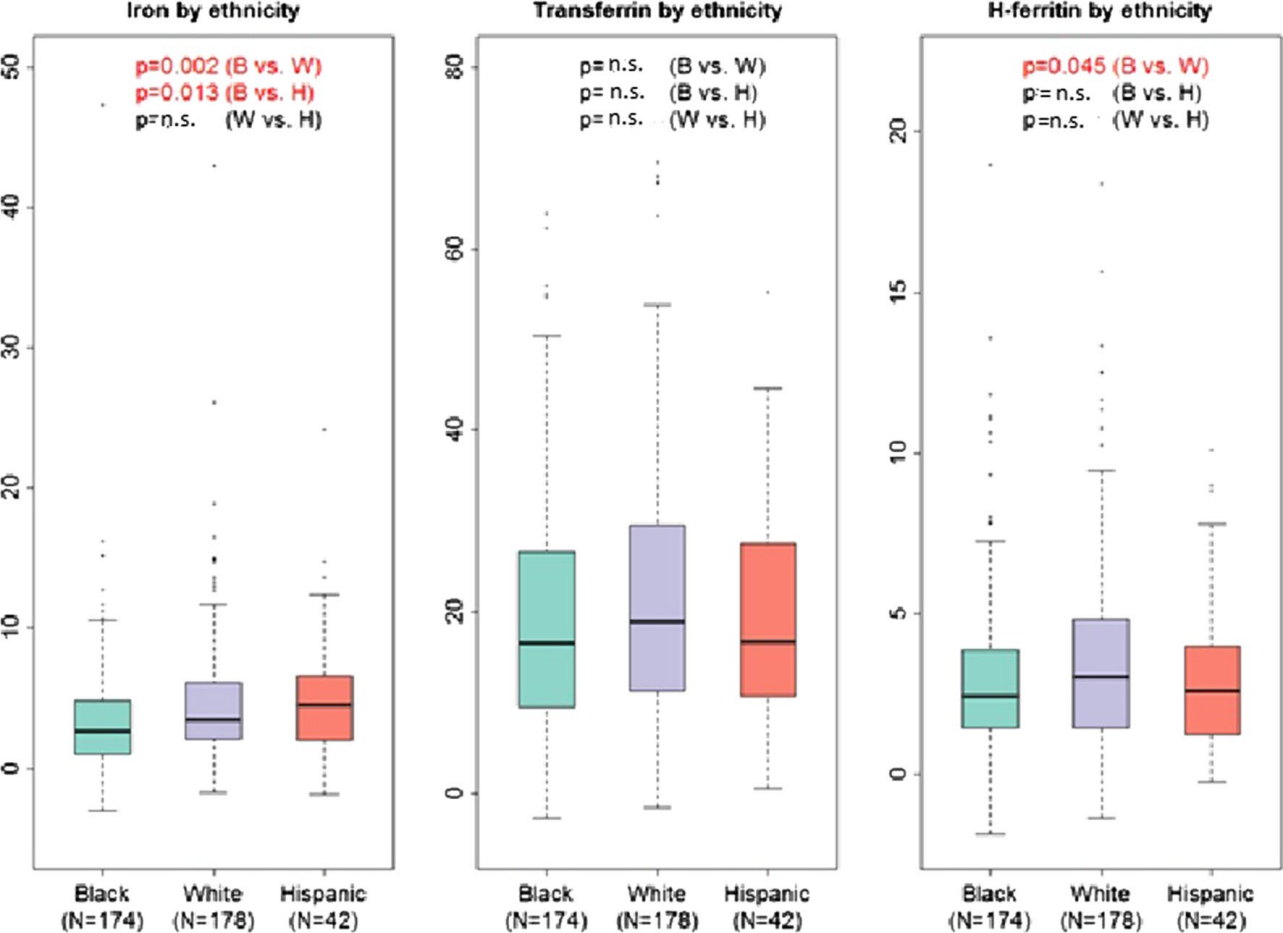
Boxplots of CSF Iron biomarker distributions in CHARTER study participants based on self-reported race and ethnicity. *p*-values <0.05 are indicated in *red*. Units of measurement are as: iron (µg/dL), transferrin (µg/mL), H-ferritin (ng/mL). *B* Black, *W* White, *H* Hispanic self-reported race/ethnicity

Since there is evidence of genetic differences among population subgroups with regard to iron metabolism [66], we also compared levels of CSF iron, transferrin, and H-ferritin between self-reported HIV+ White, Black, and Hispanic study participants and were able to discern ancestry-related differences. As shown in Table 4 and Fig. 4, differences in CSF transferrin levels did not attain statistical significance between any of these subgroups in unadjusted analyses, although self-identified Whites tended to have higher CSF transferrin levels than Blacks (*p* = 0.057). CSF H-ferritin levels were significantly lower in Blacks than in Whites (*p* < 0.05) in this HIV+ sample, however, and total iron CSF iron levels were also significantly lower in Blacks compared to either Whites or Hispanics (*p* = 0.002 and *p* = 0.013, respectively).

**Table 4:** Distributions of iron biomarkers in CSF in different CHARTER subpopulations

CSF biomarker	Whites (N = 178) *Median (IQR)*	Blacks (N = 174) *Median (IQR)*	Hispanics (N = 42) *Median (IQR)*	*p*-value, (compared groups)
Iron (µg/dL)	3.50 (2.12, 6.13)	2.60 (1.00, 4.88)	4.57 (2.00, 6.63)	*<0.01* (W/B)^a^ n.s. (W/H)^b^ *0.01* (H/B)^c^
Transferrin (µg/mL)	18.91 (11.26, 29.53)	16.64 (9.49, 26.63)	16.71 (10.69, 27.56)	0.06 (W/B)^a^ n.s. (W/H)^b^ n.s. (H/B)^c^
H-ferritin (ng/mL)	3.02 (1.44, 4.84)	2.41 (1.42, 3.86)	2.61 (1.24, 4.00)	*0.04* (W/B)^a^ n.s. (W/H)^b^ n.s. (H/B)^c^

Self-reported ancestry (race/ethnicity) was reported as “Other” by 9 participants (*not shown*)

*p*-values < 0.05 (italics) are statistically significant

^a^Self-reported whites compared to black individuals

^b^Whites compared to Hispanic individuals

^c^Hispanics compared to black individuals

In unadjusted analyses (*data not shown*), CSF iron was not associated with any HIV disease-related variables, but CSF transferrin was associated with CSF VL and current ZDV use (both *p*-values <0.05) and tended to be higher in Whites than in Blacks (*p* = 0.05); CSF H-ferritin levels showed a borderline association with plasma VL detectability (*p* = 0.06) and current ZDV use (*p* = 0.05). In order to estimate the degree of association between CSF iron biomarkers and demographic or HIV-related factors while accounting for possible confounding by these factors, some of which promote inflammation, multivariable-adjusted linear regression analyses were conducted, using natural-log-transformed biomarker values to enforce normality. These results are shown in Table 5. CSF iron levels remained significantly associated with self-reported ancestry: iron levels were higher in Whites and Hispanics than in Blacks. CSF iron levels were not significantly related to plasma or CSF VL or virus detectability, current use of ART or ZDV, age, or sex in adjusted models. CSF transferrin associations with age (positive) and sex (lower levels in women, *β* = −0.225 [95% CI −0.445 to 0.006], *p* < 0.05) persisted following adjustment, but the association with ancestry lost significance. With regard to HIV-related factors, CSF transferrin levels remained negatively associated with ZDV use (*β* = −0.254 [95% CI −0.467, −0.040], *p* = 0.03) and increased with CSF VL (*β* = 0.297 [95% CI 0.145, 0.450], *p* < 0.01). H-ferritin levels in the CSF also remained positively associated with age and negatively associated with female sex after multivariable adjustment. Study participants with detectable plasma virus had lower CSF ferritin levels (*β* = −0.242 [95% CI −0.449, −0.035], *p* = 0.02), and those who were currently on ZDV also tended to have lower levels (*β* = −0.196 [95% CI −0.413, 0.021], *p* = 0.07); the association of transferrin with plasma VL detectability, though in the same direction, was non-significant. Higher H-ferritin levels were associated with increasing CSF VL (*β* = 0.142 [95% CI −0.002, 0.286], *p* = 0.05). Neither CSF transferrin nor H-ferritin was significantly related to race/ethnicity in multivariable-adjusted analyses. Estimates of association with age, sex, and ancestry did not appreciably change for any biomarker with full adjustment, including HIV disease factors.

**Table 5:** Multivariable-adjusted regression models associating CSF iron biomarkers with demographic and HIV disease-related factors in CHARTER study participants

Partially adjusted (age, sex, race/ethnicity)	Multivariable-adjusted *β* (95% CI)	*p*-value
CSF iron		
Age	0.004 (−0.007, 0.015)	n.s.
Sex (women vs. men)	0.102 (−0.134, 0.337)	n.s.
Race/ethnicity^a^	0.104 (0.037, 0.171)	*<0.01*
W compared to B	0.329 (0.130, 0.529)	*<0.01*
W compared to H	−0.014 (−0.318, 0.289)	n.s.
H compared to B	0.327 (−0.019, 0.674)	*0.01*

Fully adjusted (age, sex, race/ethnicity, ART, ZDV use, plasma virus detectability, and CSF VL)	Multivariable-adjusted *β*-estimate (95% CI)	*p*-value
CSF iron		
Detectable plasma virus (Y vs. N)	0.003 (−0.222, 0.230)	n.s.
CSF HIV RNA concentration (VL)	0.072 (−0.079, 0.223)	n.s.
ART (on vs. off)	0.141 (−0.149, 0.431)	n.s.
Current ZDV use (Y vs. N)	0.037 (−0.206, 0.280)	n.s.

Partially adjusted (age, sex, race/ethnicity, ZDV use)	Multivariable-adjusted *β* (95% CI)	*p*-value
CSF transferrin		
Age	0.012 (0.001, 0.022)	*0.03*
Sex (women vs. men)	−0.225 (−0.445, 0.006)	*0.04*
Race/ethnicity^a^	0.036 (−0.025, 0.097)	n.s.
W compared to B	0.110 (−0.078, 0.297)	n.s.
W compared to H	0.103 (−0.185, 0.390)	n.s.
H compared to B	0.070 (−0.233, 0.372)	n.s.
Current ZDV use (Y vs. N)	−0.254 (−0.467, −0.040)	*0.03*

Fully adjusted (age, sex, race/ethnicity, ART, ZDV use, plasma virus detectability and CSF VL)	Multivariable-adjusted *β* (95% CI)	*p*-value
CSF transferrin		
Detectable plasma virus (Y vs. N)	−0.172 (−0.376, 0.032)	n.s.
CSF HIV RNA concentration (VL)	0.297 (0.145, 0.450)	*<0.01*
ART (on vs. off)	0.153 (−0.107, 0.413)	n.s.

Partially adjusted (age, sex, race/ethnicity, ZDV use)	Multivariable-adjusted *β* (95% CI)	*p*-value
CSF ferritin		
Age	0.017 (0.006, 0.027)	*<0.01*
Sex (women vs. men)	−0.324 (−0.550, −0.097)	*<0.01*
Race/ethnicity^a^	0.020 (−0.042, 0.081)	n.s.
W compared to B	0.072 (−0.121, 0.264)	n.s.
W compared to H	0.017 (−0.297, 0.332)	n.s.
H compared to B	0.049 (−0.235, 0.333)	n.s.
Current ZDV use (Y/N)	−0.196 (−0.413, 0.021)	n.s.

Fully adjusted (age, sex, race/ethnicity, ART, ZDV use, plasma virus detectability, and CSF VL)	Multivariable-adjusted *β* (95% CI)	*p*-value
CSF ferritin		
Detectable plasma virus (Y vs. N)	−0.242 (−0.449, −0.035)	*0.02*
CSF HIV RNA concentration (VL)	0.142 (−002, 0.286)	*0.05*
ART (on vs. off)	−0.017 (−0.285, 0.251)	n.s.

The natural logarithm of each biomarker variable was used in all analyses due to non-normal distributions. For each biomarker, partially and fully adjusted separate multiple linear regression models are shown, which included the covariates listed, with *β*-estimates and their 95% confidence intervals and *p*-values. *p*-values <0.05 are statistically significant

For each biomarker, significance of covariates in partially adjusted models did not change appreciably with full adjustment

*W* Whites, *B* Blacks, *H* Hispanics (self-reported ancestry), *VL* viral (HIV RNA) load, *ZDV* zidovudine, *ART* antiretroviral therapy, *CI* confidence interval, *CSF* cerebrospinal fluid

^a^For each dichotomous comparison by race/ethnicity (ancestry), only one ancestry term (e.g., whites compared to black individuals) was entered into the model

In the subset of individuals in this study who had available Q_Alb_ data (N = 110), similar multivariable regression analyses were performed. In these models, Q_Alb_ as well as comorbidity were included as additional covariates to adjust for likely changes in the functional integrity of the blood-CSF barrier that occur with age as well as HIV infection, and for comorbidity-related inflammation, which can influence iron biomarkers [14]. These models are summarized in Table 6. Higher CSF iron was again significantly associated with race/ethnicity (*β* = 0.732, *p* = 0.02; higher iron in Hispanics and Whites than in Blacks), as well as with plasma virus detectability (*β* = 0.611, *p* = 0.05) but lower CSF VL (*β* = −0.511, *p* = 0.03). Higher transferrin levels were associated with male sex (*β* = *−*0.389, *p* = 0.03), non-White race/ethnicity (*β* = *−*0.100, *p* = 0.057, and being on ART (*β* = 0.801*, p* = 0.03), but not with comorbidity; the associations with age and ZDV use lost significance. Finally, levels of H-ferritin in CSF were significantly higher among subjects with clinically mild-to-moderate comorbidity (*β* = 0.416, *p* = 0.03) and when plasma virus was undetectable (*β* = −0.407, *p* = 0.03); CSF VL, age, and gender were no longer significant factors, though the direction of the point estimate for age remained the same. Associations for all iron biomarkers with blood–CSF barrier integrity (mean Q_Alb_) were statistically significant with the exception of H-ferritin (*p*-value = 0.07). None of the CSF iron biomarkers was associated with nadir CD4+ T-cell count, hemoglobin level, or anemia (*data not shown*).

**Table 6:** Multivariable-adjusted regression models in the subset of CHARTER study participants (N = 110) who had available, matched CSF and serum albumin measurements

Iron biomarker or covariate	Multivariable-adjusted *β* (95% CI)	*p*-value
CSF iron		
Age	−0.014 (−0.055, 0.027)	n.s.
Sex (women vs. men)	0.258 (−0.347, 0.864)	n.s.
Race/ethnicity^a^	0.732 (0.108, 1.36)	*0.02*
Detectable plasma virus (Y vs. N)	0.611 (−0.022, 1.24)	0.06^b^
Log_10_ (CSF HIV RNA concentration)	−0.581 (−1.09, −0.072)	*0.03*
ART on vs. off	0.234 (−0.544, 1.01)	n.s.
Q_Alb_	0.215 (0.023, 0.407)	*0.03*
Comorbidity (mild-moderate vs. minimal)	0.098 (−0.466, 0.661)	n.s.
CSF transferrin		
Age	0.005 (−0.016, 0.025)	n.s.
Sex (women vs. men)	−0.389 (−0.750, −0.029)	*0.03*
Race/ethnicity	−0.100 (−0.202, 0.002)	*0.06* ^b^
Detectable plasma virus (Y vs. N)	−0.322 (−0.736, 0.090)	n.s.
Log_10_ (CSF HIV RNA concentration)	0.380 (0.001, 0.760)	*0.05* ^c^
ART on *vs.* off	0.801 (0.067, 1.54)	*0.03*
Current ZDV use (Y vs. N)	0.009 (−0.364, 0.381)	n.s.
Q_Alb_	0.723 (0.346, 1.10)	*<0.01*
Comorbidity (mild-moderate vs. minimal)	0.007 (−0.299, 0.313)	n.s.
CSF H-ferritin		
Age	0.734 (−0.438, 1.91)	n.s.
Sex (women *vs.* men)	0.022 (−0.482, 0.487)	n.s.
Race/ethnicity	−0.036 (−0.378, 0.305)	n.s.
Detectable plasma virus (Y vs. N)	−0.407 (−0.780, −0.033)	*0.03*
Log_10_ (CSF HIV RNA concentration)	0.277 (−0.129, 0.683)	n.s.
ART on vs. off	0.688 (−0.197,1.57)	n.s.
Q_Alb_	0.084 (−0.006, 0.173)	n.s.
Comorbidity (mild-moderate vs. minimal)	0.416 (0.038, 0.793)	*0.03*

For each biomarker, all covariates in the regression model, including CSF: serum albumin ratio (QAlb), are shown. Biomarker values were (natural) log-transformed for all analyses

^a^Self-reported Hispanics or non-Hispanic Whites vs. Blacks

^b^ *p*-value = 0.057

^c^ *p*-value = 0.049

Iron and biomarkers of iron transport were quantified in serum in 11 individuals with available serum as well as measured CSF biomarkers at 6 months, and we evaluated correlations of iron, ferritin and transferrin levels in serum to levels of the same analytes in CSF (Additional file 3: Table S2); the methods of quantification were the same. No significant correlations were observed between levels of any of these biomarkers in CSF and their corresponding serum values.

## Discussion

This represents the first large study to systematically quantify levels of iron and the two major iron-transport proteins, transferrin and (H)-ferritin, in the CSF of HIV+ persons and to evaluate their associations with known predictors of neurocognitive impairment in this population. Due to the invasive nature of lumbar puncture, published data for CSF iron biomarkers have been scarce even in HIV-negative individuals, in whom data are available only in small numbers of individuals with specific disorders and at different stages of neurodegenerative disease [36, 37, 67–69]. Hence, normal variations of these biomarkers in healthy persons are also not well documented in the literature [38, 67, 70]. Our findings in over 400 clinically well-characterized HIV+ individuals demonstrate that CSF iron and transferrin are independently associated with demographic factors (race/ethnicity and/or sex) and HIV-disease-specific factors (plasma virus detectability, ART, and/or CSF VL), while CSF H-ferritin is related to comorbidity and virus detectability in plasma. Interestingly, observed differences in CSF iron by self-reported race/ethnicity parallel known differences in the risk of neurocognitive decline in HIV+ adults, with the highest levels occurring in persons of Hispanic ethnicity who also appear to be at increased risk of HAND [13]. This study provides the only available estimates of the range and degree of variability of iron biomarkers in HIV + CSF currently available, and the observed relationships to known risk factors for HAND are hypothesis-generating with regard to the potential role of iron dyshomeostasis in HIV-associated neurocognitive decline.

Both nonhuman primate and human studies have shown that systemic iron deficiency and anemia have lasting impact on brain iron homeostasis and neuronal health, influencing memory and cognition [32, 60, 71–73]. In Alzheimer’s disease and ALS, both peripheral and CSF iron biomarker profiles suggest disruption of iron homeostasis with concomitant inflammation within the brain [28, 30, 34, 35]. The situation in HIV infection may be similar, with neuroinflammation due to HIV-activated macrophage–monocytes migrating across a damaged BBB triggering a reduction in iron bioavailability in the CNS that may promote neurocognitive decline. Accumulating evidence for systemic changes in iron transport by HIV [54, 74, 75], mediated at least in part by the iron-regulatory hormone hepcidin, and appearance of the virus in the CNS within days of acute infection, suggest that HIV-mediated iron dysregulation may also occur in the CNS. HIV does not infect neurons, but the virus has been shown to infect perivascular macrophages, microglia, and other supporting cells within the CNS, some of which play central roles in regulating brain iron content and distribution [4, 24, 50]. HIV infection of macrophages has also been shown to downregulate expression of the MHC-class I-like iron-regulatory protein Hfe on the macrophage cell surface via the actions of viral proteins, thereby increasing macrophage iron content to benefit viral replication [76].

In the present study, higher CSF iron content tended to be associated with detectable virus in the plasma but with lower CSF VL, when adjusting for the Q_Alb_, a measure reflecting variability in integrity of the blood–CSF barrier. This apparent discordance suggests that either (1) more iron fails to cross the CSF–brain barrier when HIV levels in the brain or CSF are low, leading to higher levels in the CSF, or (2) higher amounts of virus in the brain or CSF are associated with greater extraction of iron from the CSF, because HIV requires iron for its replication [77]. The significantly lower CSF transferrin levels among persons of Hispanic ethnicity and non-Hispanic Whites compared to Blacks after adjustment for Q_Alb_ were also in accord with the corresponding observed CSF iron differences. Even when accounting for the effects of ZDV, an antiretroviral drug that can affect iron transport, ART was independently associated with higher CSF transferrin, raising the possibility that other commonly used antiretroviral drugs may impact transferrin levels in the CNS, possibly by inducing a functionally iron-deficient state. Initiation of ART has been temporally linked to an increase in soluble transferrin receptors, a sensitive indicator of tissue iron deficiency [78, 79]. Other mechanisms by which functional iron deficiency might be induced in the CNS during HIV infection are the depletion of cellular iron due to active viral replication and/or chronic inflammation in the brain that results in iron entrapment within supporting cells that normally deliver iron, or iron deficiency of these cells themselves [24, 76, 80]. Indeed, higher CSF VL was associated in our analyses with higher CSF transferrin levels, with and without Q_Alb_ adjustment. Interestingly, HIV+ individuals are at increased risk of RLS, a condition associated with iron deficiency in the brain [81, 82].

Although men are known to have significantly higher systemic iron stores than pre-menopausal women, differences in CSF levels of iron, transferrin, or H-ferritin levels have not been documented, and they may have clinical relevance [83]. Women were observed to have significantly lower levels of both transferrin and H-ferritin before Q_Alb_ adjustment; this association persisted following adjustment only for transferrin, and the direction of the estimate remained the same for H-ferritin although significance was lost. That higher H-ferritin levels were associated with undetectable plasma virus but a higher quantity of CSF virus suggests that induction of H-ferritin in the CNS may be a physiological (cytoprotective) response to viral compartmentalization and replication in the CNS. HIV-induced oxidative stress is manifested by upregulation of heme-oxygenase-1 in the brain [84], and the ferroxidase activity of H-ferritin has recently been shown to mediate the antioxidant activity of heme oxygenase-1 [85]. Due to its high metabolic demands, the brain also requires a steady supply of iron in non-reactive form, for energy production as well as myelin and neurotransmitter synthesis [61, 86–88]. Todorich et al. [46] previously identified H-ferritin as an important source of iron for oligodendrocytes, and although the origin of CSF iron, H-ferritin, and transferrin cannot be established in this type of study, it is possible that both proteins are upregulated in persons with suppressed viremia in an attempt to preserve iron delivery to oligodendrocytes, other supporting cells of the CNS, and neurons. Indeed, HIV infection is associated with decreased amounts of myelin or loss of myelin integrity [89–91]. One neuroimaging study that used modern, iron-sensitive techniques reported possible decreases in brain iron in HIV-infected individuals [40], and studies using diffusion tensor imaging or other modalities have consistently revealed white matter damage [40, 83]. It is not surprising that CSF H-ferritin levels were also associated with more clinically significant comorbidity, as comorbidity has emerged as a powerful predictor of neurocognitive decline and is a common correlate of chronic inflammation in HIV+ persons [92]. Mechanisms other than inflammation may also explain increases in H-ferritin in CSF, including HIV-induced changes in autophagy or even ferritinophagy [93, 94]. Finally, associations with age, a strong risk factor for HAND, with both CSF transferrin and H-ferritin were pronounced before adjusting for Q_Alb_ but lost significance after this adjustment; this begs the question whether the loss of iron regulation (or the loss of compartmentalization of iron) in the CSF due to age- or HIV-related damage to the blood–CSF barrier may in part mediate age-related neurocognitive decline.

Levels of CSF iron biomarkers in this HIV+ population were not dramatically different from levels previously reported in small numbers of healthy individuals, but the different assay methods used complicate any comparisons: CSF ferritin levels in this study were lower than levels in individuals either with or without RLS that were reported by Mizuno et al. and they were also lower than levels observed in chronic demyelinating disorders like chronic progressive multiple sclerosis [37, 38, 67]. Assays for total ferritin, rather than H-ferritin, may also have been used in previous studies. As with other intracellular microorganisms, the manipulation of cellular iron homeostasis by HIV to benefit its own replication may eventually result in altered brain iron balance (either functional iron deficiency, or iron excess in specific brain regions, or a combination of these). Significant changes in total CSF iron biomarker levels may or may not accurately reflect these regional changes. CSF iron, transferrin and ferritin did not appear to be correlated with their respective serum levels in 11 individuals with visit-matched CSF and serum samples, but the small size of the sample with both sets of measurements precludes firm conclusions. While possibly due to insufficient power, the absence of significant correlations supports the concept that levels of iron and iron transporters in the CNS are actively regulated and do not merely reflect passive influx from the circulation; this view is entirely consistent with prior studies [50]. Differences in serum and CSF assays used for iron-biomarker quantification and the fact that H-ferritin was measured in CSF, while the L subunit (iron-poor form) of ferritin predominates in serum, could also account for these differences. Relatively little is known about mechanisms controlling total or H-ferritin levels in either compartment [95].

We acknowledge the limitations of this study, including its cross-sectional design, which limits interpretation, the lack of an HIV-negative comparison group, and absence of matched serum iron biomarker data for most participants. Moderately correlated, within-individual values of iron, transferrin and H-ferritin obtained at baseline and 6 months in 100 of the study participants, however, support the use of single CSF biomarker measurements for our analyses and favor their stability in HIV+ persons over short periods of time. Regarding the lack of HIV-negative controls, we would stress that our objective was not to determine the impact of HIV on CSF iron biomarker levels, but to ascertain their associations with key demographic, HIV disease- and treatment-related predictors of neurocognitive decline, since measures of HAND are often imprecise and fluctuate in individuals over time [4]. Better knowledge of iron biomarker variability and associations with these other host factors will facilitate the design of future studies and inform the precision of predictive models. Longitudinal follow-up studies with measurements of biomarkers at more than one time-point in individual disease trajectories will be needed to clarify the temporal sequence of iron dysregulation after HIV infection, although such studies may be challenging due to the invasive nature of CSF sampling. This study provides a systematic assessment of the demographic and disease-related factors associated with CSF iron biomarker levels in HIV+ persons, laying the foundation for studies that specifically evaluate whether changes in these iron-related measures promote the development of HAND.

## Conclusions

In HIV+ adults, CSF levels of iron and major biomarkers of iron-transport are independently associated with sex, self-reported race/ethnicity, ART, plasma virus detectability and/or CSF VL. Associations of CSF iron biomarkers with age, which disappeared following adjustment for a measure of blood-CSF barrier function, suggests the possibility that age- or HIV-related declines in the integrity of the blood-CSF barrier may contribute to changes in CNS iron balance as HIV+ individuals age. Furthermore, this study raises the intriguing possibility that consistent correlates of HAND such as race/ethnicity, ART, and HIV RNA in the CSF, may be linked to HAND in part via changes in CNS iron homeostasis. Additional studies, preferably including longitudinal samples and a higher number of participants over 50 years of age, as well as matched HIV-negative controls, are needed to further investigate these questions.

## Additional files

**Additional file 1: Figure S1.** Scatter plots of iron biomarker values in CSF at baseline (N=403) and 6 months (N=100). *P*-values shown are for corresponding Spearman correlations.

**Additional file 2: Table S1.** Iron biomarker values for CHARTER study participants at baseline and 6-months. Units of measurement: (CSF) iron, µg/dL; transferrin, µg/mL; ferritin, ng/mL.

**Additional file 3.** Visit-matched (6-month) CSF and serum iron biomarker values for 11 CHARTER study participants. Units of measurement: (CSF) iron, µg/dL; transferrin, µg/mL; ferritin, ng/mL; (Serum) iron, µg/dL; transferrin, mg/dL; ferritin, ng/mL.

## Abbreviations

CSF: cerebrospinal fluid
ART: antiretroviral therapy
HAND: HIV-associated neurocognitive disorder
CNS: central nervous system
HIV+: HIV-infected
ZDV: zidovudine
ALS: amyotrophic lateral sclerosis
RLS: restless leg syndrome
BBB: blood–brain barrier
VL: viral load (HIV RNA concentration)
CHARTER: CNS HIV antiretroviral therapy effects research (study)
IQR: interquartile range
Q_Alb_: CSF to serum albumin ratio
