# Supplementary material for: Cerebrospinal fluid (CSF) biomarkers of iron status are associated with CSF viral load, antiretroviral therapy, and demographic factors in HIV-infected adults
Source: Fluids Barriers CNS. 2017 Apr 21;14:11. doi: 10.1186/s12987-017-0058-1 (PMC5399327; doi:10.1186/s12987-017-0058-1)
Supplement: Supplementary file 1 — Additional file 1: Figure S1. Scatter plots of iron biomarker values in CSF at baseline (N=403) and 6 months (N=100). P-values shown are for corresponding Spearman correlations. [file 12987_2017_58_MOESM1_ESM.docx]

0

20

40

60

80

100

Second Measurement

µg/mL

0

20

40

60

80

First Measurement

*p-value<0.0001*

Transferrin

0

5

10

15

20

Second Measurement

µg/dL

0

10

20

30

40

50

First Measurement

*p-value<0.01*

Iron

0

5

10

15

20

Second Measurement

ng/mL

0

5

10

15

20

First Measurement

ferritin_6

*p-value<0.0001*

Ferritin
