# Supplementary material for: Cerebrospinal fluid (CSF) biomarkers of iron status are associated with CSF viral load, antiretroviral therapy, and demographic factors in HIV-infected adults
Source: Fluids Barriers CNS. 2017 Apr 21;14:11. doi: 10.1186/s12987-017-0058-1 (PMC5399327; doi:10.1186/s12987-017-0058-1)
Supplement: Supplementary file 2 — Additional file 2: Table S1. Iron biomarker values for CHARTER study participants at baseline and 6-months. Units of measurement: (CSF) iron, µg/dL; transferrin, µg/mL; ferritin, ng/mL. [file 12987_2017_58_MOESM2_ESM.docx]

| **Patient** | **Baseline CSF measurement**  **(N=403)** | | | **6-month CSF measurement**  **(N=100)** | | |
| --- | --- | --- | --- | --- | --- | --- |
|  | **Iron** | **Transferrin** | **Ferritin** | **Iron** | **Transferrin** | **Ferritin** |
| 1 | 7.4 | 8.8 | 6.8 | 9.3 | 10.3 | 6.6 |
| 2 | 6.4 | 14.3 | 18.4 | 4.4 | 16.0 | 16 |
| 3 | 1.8 | 15.0 | 4.2 | 1.2 | 10.2 | 2.2 |
| 4 | 5.3 | 4.9 | 5.1 | 0.3 | 6.2 | 4.7 |
| 5 | 0.7 | 7.9 | 2.8 | 0 | 3.2 | 8.4 |
| 6 | 0 | 11.4 | 5.4 | 3.9 | 10.8 | 6.3 |
| 7 | 8.2 | 13.0 | 15.7 | 7.7 | 11.5 | 11.3 |
| 8 | 4.3 | 12.0 | 8.0 | 3.4 | 8.9 | 7.5 |
| 9 | 5.9 | 15.7 | 6.3 | 4.8 | 9.1 | 5.2 |
| 10 | 4.5 | 14.9 | 2.6 | 0 | 12.5 | 7.1 |
| 11 | 4.0 | 11.3 | 3.7 | 0.2 | 12.7 | 5.1 |
| 12 | 43 | 3.9 | 6.3 | 1.8 | 15.5 | 2.7 |
| 13 | 0.9 | 14.3 | 6.1 | 4.8 | 14.3 | 7.3 |
| 14 | 15.0 | 10.7 | 9.0 | 1.3 | 15.2 | 3.8 |
| 15 | 12.8 | 7.4 | 2.2 | 3.4 | 3.2 | 7.2 |
| 16 | 4.7 | 15.0 | 9.5 | 3.9 | 11.8 | 2.8 |
| 17 | 1.5 | 11.3 | 3.4 | 2.4 | 9.5 | 2.9 |
| 18 | 26.2 | 11.3 | 5.5 | 2.9 | 15.1 | 0.8 |
| 19 | 2.3 | 1.3 | 1.5 | 0.8 | 10.8 | 10.7 |
| 20 | 4.5 | 5.5 | 5.5 | 7.5 | 10.2 | 15.6 |
| 21 | 2.4 | 29.6 | 0 | 3.9 | 38.5 | 0.5 |
| 22 | 9.5 | 13.2 | 2.4 | 4.5 | 38.0 | 5.4 |
| 23 | 5.3 | 27.2 | 9.0 | 1.8 | 25.3 | 5.0 |
| 24 | 2.7 | 36.3 | 5.3 | 1.7 | 28.9 | 2.8 |
| 25 | 2.4 | 67.5 | 3.0 | 8.8 | 57.7 | 0.7 |
| 26 | 4.0 | 27.3 | 2.0 | 16.2 | 18.3 | 1.9 |
| 27 | 5.5 | 40.8 | 2.6 | 3.2 | 52.4 | 5.0 |
| 28 | 3.4 | 25.1 | 8.7 | 4.5 | 37.6 | 6.2 |
| 29 | 5.7 | 32.3 | 12.5 | 5.5 | 51.3 | 6.9 |
| 30 | 4.0 | 19.9 | 4.6 | 5.7 | 36.4 | 8.5 |
| 31 | 6.0 | 27.6 | 3.9 | 1.8 | 44.8 | 4.4 |
| 32 | 3.7 | 36.4 | 0.8 | 10.3 | 58.0 | 5.6 |
| 33 | 4.9 | 31.3 | 3.4 | 4.4 | 30.3 | 5.0 |
| 34 | 4.9 | 55.1 | 1.7 | 5.7 | 50.6 | 6.9 |
| 35 | 0.5 | 13.3 | 2.4 | 2.0 | 32.3 | 1.9 |
| 36 | 1.4 | 29.6 | 7.8 | 1.7 | 51.4 | 8.8 |
| 37 | 2.5 | 17.5 | 6.1 | 3.8 | 27.4 | 4.2 |
| 38 | 7.0 | 69.6 | 2.0 | 2.4 | 59.7 | 4.6 |
| 39 | 3.2 | 21.3 | 0.2 | 1.3 | 23.5 | 1.3 |
| 40 | 3.9 | 14.8 | 3.4 | 2.8 | 21 | 1.7 |
| 41 | 5.5 | 27.1 | 2.9 | 5.7 | 19.8 | 4.6 |
| 42 | 11.3 | 15.2 | 5.1 | 10.2 | 67.5 | 5.1 |
| 43 | 9.9 | 16.9 | 7.8 | 4.7 | 26.3 | 1.1 |
| 44 | 3.5 | 10.8 | 2.0 | 3.3 | 11.8 | 2.2 |
| 45 | 0.7 | 15.5 | 3.1 | 0 | 0 | 0.3 |
| 46 | 1.7 | 40.5 | 7.3 | 3.5 | 35.0 | 5.3 |
| 47 | 4.2 | 38.2 | 2.7 | 3.8 | 58.7 | 1.9 |
| 48 | 1.9 | 67.4 | 0.8 | 3.4 | 38.0 | 1.4 |
| 49 | 6.7 | 55.3 | 4.8 | 8.9 | 15.6 | 2.7 |
| 50 | 2.3 | 41.5 | 2.8 | 2.9 | 44.6 | 1 |
| 51 | 2.5 | 24.7 | 4.7 | 0.3 | 19.6 | 2.4 |
| 52 | 0 | 4.2 | 1.5 | 0.7 | 31.7 | 0.2 |
| 53 | 1.9 | 36.5 | 2.8 | 1.3 | 69.4 | 0.8 |
| 54 | 1.7 | 46.8 | 4.0 | 9.9 | 84.2 | 3.1 |
| 55 | 4.0 | 20.0 | 2.3 | 6.3 | 7.8 | 0.8 |
| 56 | 6.4 | 34.9 | 0.9 | 4.0 | 66.6 | 1.2 |
| 57 | 5.5 | 31.8 | 10.1 | 6.9 | 21.4 | 6.9 |
| 58 | 3.9 | 40.9 | 1.6 | 4.8 | 58.6 | 1.4 |
| 59 | 2.2 | 0 | 0 | 2.9 | 29.9 | 2.2 |
| 60 | 2.8 | 5.8 | 3.8 | 3.7 | 1.2 | 6 |
| 61 | 5.2 | 5.8 | 2.2 | 6.5 | 17.0 | 3.2 |
| 62 | 5.3 | 14.0 | 1.5 | 3.7 | 0 | 1.2 |
| 63 | 3.9 | 10.7 | 1.8 | 3.0 | 19.8 | 1.8 |
| 64 | 2.7 | 10.1 | 1.0 | 3.7 | 8.1 | 1.7 |
| 65 | 2.9 | 16.5 | 1.3 | 1.5 | 13.8 | 1.6 |
| 66 | 1.4 | 19.5 | 3.2 | 2.7 | 14.0 | 4.4 |
| 67 | 4.8 | 15.6 | 1.4 | 6.8 | 12.2 | 6.2 |
| 68 | 4.3 | 2.7 | 0 | 5.5 | 10.6 | 0 |
| 69 | 4.4 | 19.5 | 2.3 | 5.8 | 16.0 | 0 |
| 70 | 3.0 | 6.7 | 0.5 | 4.4 | 0 | 0 |
| 71 | 3.4 | 8.4 | 1.1 | 0 | 3.4 | 0.4 |
| 72 | 0 | 3.8 | 0 | 1.3 | 9.7 | 0.4 |
| 73 | 2.2 | 19.3 | 0.7 | 10.8 | 13.5 | 4.8 |
| 74 | 2.4 | 10.3 | 5.5 | 0 | 2.2 | 0 |
| 75 | 2.4 | 8.4 | 1.0 | 2.0 | 0 | 0 |
| 76 | 3.5 | 18.4 | 0 | 3.5 | 15.2 | 2.8 |
| 77 | 1.2 | 0 | 0 | 1.9 | 5.1 | 0.4 |
| 78 | 3.9 | 9.1 | 3.3 | 8.2 | 13.6 | 3.6 |
| 79 | 2.7 | 8.0 | 0 | 6.4 | 9.9 | 0 |
| 80 | 24.2 | 22.8 | 7.8 | 7.8 | 27.1 | 6.2 |
| 81 | 0.5 | 7.5 | 1.3 | 3.3 | 11.1 | 3 |
| 82 | 3.0 | 9.4 | 2.1 | 7.0 | 16.7 | 2.7 |
| 83 | 2.9 | 15.6 | 2.4 | 0 | 15.4 | 2.1 |
| 84 | 6.2 | 15.1 | 2.9 | 0.3 | 12.7 | 1.3 |
| 85 | 3.3 | 16.6 | 3.2 | 2.0 | 15.2 | 3.0 |
| 86 | 0 | 5.4 | 2.3 | 3.4 | 12.8 | 2.2 |
| 87 | 2.8 | 7.9 | 1.5 | 1.9 | 10.9 | 1.8 |
| 88 | 0 | 8.9 | 0.8 | 7.9 | 12.1 | 2.7 |
| 89 | 2.3 | 20.5 | 4.0 | 1.5 | 21.0 | 5.0 |
| 90 | 0 | 8.2 | 2.4 | 1.3 | 13.9 | 1.5 |
| 91 | 1.9 | 0.9 | 1.9 | 0 | 0.7 | 2.1 |
| 92 | 1.0 | 14.1 | 3.9 | 2.9 | 16.8 | 1.9 |
| 93 | 1.9 | 13.0 | 3.2 | 3.7 | 17.9 | 6.1 |
| 94 | 0 | 11.5 | 2.5 | 0 | 8.6 | 2.4 |
| 95 | 0 | 16.6 | 3.6 | 1.4 | 23.7 | 4.1 |
| 96 | 6.4 | 0.6 | 0.7 | 0 | 10.1 | 1.4 |
| 97 | 0 | 10.5 | 1.3 | 0 | 8.5 | 1.2 |
| 98 | 2.2 | 14.0 | 3.4 | 9.3 | 16.7 | 2.5 |
| 99 | 2.0 | 12.5 | 3.3 | 1.4 | 0 | 4.6 |
| 100 | 3.7 | 15.8 | 4.6 | 3.2 | 16.8 | 2 |
| 101 | 3.3 | 19.1 | 3.1 | --- | --- | --- |
| 102 | 7.5 | 5.4 | 11.4 | --- | --- | --- |
| 103 | 6.8 | 21.4 | 2.1 | --- | --- | --- |
| 104 | 6.8 | 9.1 | 2.9 | --- | --- | --- |
| 105 | 11.2 | 18.5 | 4.6 | --- | --- | --- |
| 106 | 3.4 | 14.6 | 4.9 | --- | --- | --- |
| 107 | 10.9 | 15.6 | 2.1 | --- | --- | --- |
| 108 | 5.0 | 2.9 | 1.8 | --- | --- | --- |
| 109 | 3.2 | 14.7 | 3.6 | --- | --- | --- |
| 110 | 1.8 | 12.8 | 3.1 | --- | --- | --- |
| 111 | 1.2 | 10.4 | 3.3 | --- | --- | --- |
| 112 | 2.5 | 10.6 | 3.2 | --- | --- | --- |
| 113 | 5.9 | 21.4 | 4.9 | --- | --- | --- |
| 114 | 4.4 | 18.1 | 3.3 | --- | --- | --- |
| 115 | 4.2 | 17.9 | 5.9 | --- | --- | --- |
| 116 | 3.3 | 12.3 | 1.5 | --- | --- | --- |
| 117 | 7.7 | 20.4 | 6.8 | --- | --- | --- |
| 118 | 7.3 | 24.2 | 8.7 | --- | --- | --- |
| 119 | 5.9 | 21.9 | 4.9 | --- | --- | --- |
| 120 | 1.8 | 13.1 | 2.3 | --- | --- | --- |
| 121 | 1.5 | 3.2 | 1.4 | --- | --- | --- |
| 122 | 2.7 | 16.6 | 6.9 | --- | --- | --- |
| 123 | 5.7 | 11 | 2.4 | --- | --- | --- |
| 124 | 2.5 | 8.9 | 2.7 | --- | --- | --- |
| 125 | 3.5 | 4.8 | 4.2 | --- | --- | --- |
| 126 | 6.4 | 6.8 | 5.8 | --- | --- | --- |
| 127 | 6.2 | 9.5 | 5.2 | --- | --- | --- |
| 128 | 3.2 | 13.3 | 4.2 | --- | --- | --- |
| 129 | 1.8 | 17.9 | 4.1 | --- | --- | --- |
| 130 | 5.5 | 16.5 | 3.2 | --- | --- | --- |
| 131 | 5.9 | 22.3 | 4.9 | --- | --- | --- |
| 132 | 4.0 | 20.1 | 5.5 | --- | --- | --- |
| 133 | 2.0 | 21.7 | 3.8 | --- | --- | --- |
| 134 | 2.8 | 11.8 | 4.3 | --- | --- | --- |
| 135 | 2.9 | 14.3 | 2.3 | --- | --- | --- |
| 136 | 3.2 | 19.9 | 6.7 | --- | --- | --- |
| 137 | 1.8 | 13.1 | 2.0 | --- | --- | --- |
| 138 | 1.2 | 14.3 | 1.9 | --- | --- | --- |
| 139 | 4.9 | 6.3 | 2.8 | --- | --- | --- |
| 140 | 6.3 | 20.0 | 3.1 | --- | --- | --- |
| 141 | 6.0 | 10.9 | 4.8 | --- | --- | --- |
| 142 | 2.1 | 15.3 | 1.5 | --- | --- | --- |
| 143 | 4.1 | 14.3 | 0 | --- | --- | --- |
| 144 | 7.5 | 13.7 | 1.5 | --- | --- | --- |
| 145 | 1.2 | 7.0 | 0.4 | --- | --- | --- |
| 146 | 0.8 | 17.1 | 0.3 | --- | --- | --- |
| 147 | 3.4 | 22.8 | 3.7 | --- | --- | --- |
| 148 | 0.8 | 7.3 | 0.9 | --- | --- | --- |
| 149 | 1.6 | 12.5 | 0 | --- | --- | --- |
| 150 | 5.7 | 14.2 | 5.7 | --- | --- | --- |
| 151 | 0 | 8.0 | 1.2 | --- | --- | --- |
| 152 | 4.1 | 15.6 | 3.4 | --- | --- | --- |
| 153 | 2.8 | 0 | 4.9 | --- | --- | --- |
| 154 | 4.8 | 17.8 | 4.2 | --- | --- | --- |
| 155 | 0.8 | 12.9 | 0.9 | --- | --- | --- |
| 156 | 1.1 | 12.0 | 1.0 | --- | --- | --- |
| 157 | 7.1 | 12.3 | 3.6 | --- | --- | --- |
| 158 | 14.7 | 12.4 | 0.9 | --- | --- | --- |
| 159 | 8.2 | 3.8 | 0 | --- | --- | --- |
| 160 | 11.7 | 9.1 | 0.4 | --- | --- | --- |
| 161 | 4.1 | 9.5 | 0 | --- | --- | --- |
| 162 | 1.6 | 3.1 | 0 | --- | --- | --- |
| 163 | 0 | 7.8 | 0.2 | --- | --- | --- |
| 164 | 1.7 | 9.8 | 0.6 | --- | --- | --- |
| 165 | 3.0 | 8.6 | 2.0 | --- | --- | --- |
| 166 | 4.0 | 18.4 | 1.2 | --- | --- | --- |
| 167 | 2.9 | 18.9 | 2.5 | --- | --- | --- |
| 168 | 5.6 | 10.8 | 1.6 | --- | --- | --- |
| 169 | 16.2 | 14.5 | 1.2 | --- | --- | --- |
| 170 | 2.6 | 17.1 | 7.3 | --- | --- | --- |
| 171 | 0.7 | 15.5 | 0.3 | --- | --- | --- |
| 172 | 0 | 3.6 | 0.7 | --- | --- | --- |
| 173 | 1.0 | 5.6 | 1.7 | --- | --- | --- |
| 174 | 3.2 | 7.1 | 0.9 | --- | --- | --- |
| 175 | 2.6 | 12 | 13.6 | --- | --- | --- |
| 176 | 2.7 | 17 | 5.3 | --- | --- | --- |
| 177 | 2.1 | 23.6 | 2.9 | --- | --- | --- |
| 178 | 2.9 | 11.2 | 0.5 | --- | --- | --- |
| 179 | 0.6 | 13.6 | 0 | --- | --- | --- |
| 180 | 7.5 | 0.7 | 0.9 | --- | --- | --- |
| 181 | 7.5 | 50.4 | 3.1 | --- | --- | --- |
| 182 | 0.3 | 50.5 | 3.1 | --- | --- | --- |
| 183 | 2.5 | 16.9 | 4.8 | --- | --- | --- |
| 184 | 14.9 | 23.2 | 4.7 | --- | --- | --- |
| 185 | 3.3 | 11.5 | 4.4 | --- | --- | --- |
| 186 | 0.3 | 18.7 | 11.1 | --- | --- | --- |
| 187 | 2.6 | 2.4 | 0 | --- | --- | --- |
| 188 | 0 | 19.3 | 3.3 | --- | --- | --- |
| 189 | 0 | 3.5 | 1.0 | --- | --- | --- |
| 190 | 0.4 | 16.5 | 2.7 | --- | --- | --- |
| 191 | 0 | 7.7 | 2.1 | --- | --- | --- |
| 192 | 0 | 27.6 | 1.9 | --- | --- | --- |
| 193 | 0 | 19.7 | 0.4 | --- | --- | --- |
| 194 | 0 | 14.5 | 1.9 | --- | --- | --- |
| 195 | 0 | 15.7 | 1.2 | --- | --- | --- |
| 196 | 4.3 | 25.6 | 2.5 | --- | --- | --- |
| 197 | 3.3 | 32.8 | 1.6 | --- | --- | --- |
| 198 | 11.0 | 0.2 | 0.5 | --- | --- | --- |
| 199 | 3.5 | 33.4 | 3.1 | --- | --- | --- |
| 200 | 0 | 38.3 | 0.5 | --- | --- | --- |
| 201 | 4.1 | 20.6 | 7.9 | --- | --- | --- |
| 202 | 0.6 | 27.0 | 3.9 | --- | --- | --- |
| 203 | 1.4 | 27.3 | 3.6 | --- | --- | --- |
| 204 | 0.6 | 10.1 | 2.8 | --- | --- | --- |
| 205 | 2.3 | 24 | 3.5 | --- | --- | --- |
| 206 | 1.0 | 5.1 | 5.4 | --- | --- | --- |
| 207 | 2.4 | 17 | 1.3 | --- | --- | --- |
| 208 | 5.4 | 53.9 | 8.4 | --- | --- | --- |
| 209 | 7.8 | 10.7 | 0 | --- | --- | --- |
| 210 | 0 | 0.4 | 0 | --- | --- | --- |
| 211 | 0 | 1.3 | 0.6 | --- | --- | --- |
| 212 | 7.1 | 12.6 | 2.5 | --- | --- | --- |
| 213 | 0 | 9.5 | 5.8 | --- | --- | --- |
| 214 | 0 | 6.3 | 0.5 | --- | --- | --- |
| 215 | 0 | 20.7 | 5.5 | --- | --- | --- |
| 216 | 0 | 16.1 | 0.3 | --- | --- | --- |
| 217 | 0 | 68.1 | 10.9 | --- | --- | --- |
| 218 | 4.7 | 22.0 | 5.6 | --- | --- | --- |
| 219 | 2.2 | 35.8 | 19.0 | --- | --- | --- |
| 220 | 0 | 23.8 | 2.8 | --- | --- | --- |
| 221 | 0 | 20.4 | 1.9 | --- | --- | --- |
| 222 | 2.7 | 30.3 | 7.3 | --- | --- | --- |
| 223 | 3.7 | 30.2 | 1.4 | --- | --- | --- |
| 224 | 1.9 | 3.0 | 1.0 | --- | --- | --- |
| 225 | 0.3 | 10.8 | 3.8 | --- | --- | --- |
| 226 | 4.8 | 16.8 | 1.5 | --- | --- | --- |
| 227 | 4.9 | 20.3 | 1.8 | --- | --- | --- |
| 228 | 0.7 | 25.2 | 2.7 | --- | --- | --- |
| 229 | 1.3 | 10.9 | 4.1 | --- | --- | --- |
| 230 | 0.3 | 43.3 | 3.0 | --- | --- | --- |
| 231 | 0 | 11.2 | 1.8 | --- | --- | --- |
| 232 | 0.8 | 38.1 | 3.9 | --- | --- | --- |
| 233 | 1.6 | 32.3 | 3.6 | --- | --- | --- |
| 234 | 1.0 | 41.1 | 1.8 | --- | --- | --- |
| 235 | 0 | 33.1 | 2.2 | --- | --- | --- |
| 236 | 0.4 | 21.9 | 3.9 | --- | --- | --- |
| 237 | 2.6 | 25.2 | 1.7 | --- | --- | --- |
| 238 | 3.3 | 49.2 | 4.2 | --- | --- | --- |
| 239 | 4.1 | 29.6 | 3.6 | --- | --- | --- |
| 240 | 0.8 | 26.7 | 5.9 | --- | --- | --- |
| 241 | 2.1 | 39.0 | 2.1 | --- | --- | --- |
| 242 | 1.3 | 26.6 | 4.1 | --- | --- | --- |
| 243 | 3.1 | 30.7 | 4.1 | --- | --- | --- |
| 244 | 6.1 | 27.5 | 4.7 | --- | --- | --- |
| 245 | 2.4 | 54.7 | 11.9 | --- | --- | --- |
| 246 | 3.0 | 25.2 | 2.7 | --- | --- | --- |
| 247 | 2.8 | 30.5 | 2.5 | --- | --- | --- |
| 248 | 0 | 18.7 | 2.1 | --- | --- | --- |
| 249 | 15.2 | 30.5 | 3.1 | --- | --- | --- |
| 250 | 1.5 | 2.9 | 3.3 | --- | --- | --- |
| 251 | 1.9 | 17.9 | 2.1 | --- | --- | --- |
| 252 | 3.7 | 7.8 | 2.2 | --- | --- | --- |
| 253 | 4.8 | 63.9 | 11.2 | --- | --- | --- |
| 254 | 2.5 | 62.4 | 5.2 | --- | --- | --- |
| 255 | 3.9 | 29.2 | 1.6 | --- | --- | --- |
| 256 | 2.2 | 25.7 | 0.7 | --- | --- | --- |
| 257 | 5.6 | 4.4 | 1.6 | --- | --- | --- |
| 258 | 4.5 | 15.5 | 3.1 | --- | --- | --- |
| 259 | 1.3 | 22.0 | 2.6 | --- | --- | --- |
| 260 | 0.3 | 17.2 | 1.4 | --- | --- | --- |
| 261 | 47.4 | 36.3 | 10.7 | --- | --- | --- |
| 262 | 11.8 | 37.2 | 4.8 | --- | --- | --- |
| 263 | 6.8 | 25.1 | 6.5 | --- | --- | --- |
| 264 | 8.5 | 4.7 | 7.9 | --- | --- | --- |
| 265 | 4.5 | 6.3 | 5.0 | --- | --- | --- |
| 266 | 6.0 | 25.3 | 2.1 | --- | --- | --- |
| 267 | 3.7 | 28.8 | 2.0 | --- | --- | --- |
| 268 | 1.3 | 10.3 | 1.7 | --- | --- | --- |
| 269 | 3.2 | 21.8 | 1.5 | --- | --- | --- |
| 270 | 10.4 | 63.7 | 4.8 | --- | --- | --- |
| 271 | 3.4 | 28.7 | 2.2 | --- | --- | --- |
| 272 | 3.4 | 11.5 | 1.8 | --- | --- | --- |
| 273 | 18.9 | 39.1 | 4.1 | --- | --- | --- |
| 274 | 7.3 | 18.4 | 3.8 | --- | --- | --- |
| 275 | 5.2 | 37.2 | 10.3 | --- | --- | --- |
| 276 | 6.4 | 36.8 | 3.2 | --- | --- | --- |
| 277 | 8.3 | 32.5 | 8.9 | --- | --- | --- |
| 278 | 11.0 | 52.9 | 4.1 | --- | --- | --- |
| 279 | 9.0 | 2.5 | 3.0 | --- | --- | --- |
| 280 | 2.0 | 10.3 | 1.7 | --- | --- | --- |
| 281 | 13.0 | 31.0 | 3.0 | --- | --- | --- |
| 282 | 6.3 | 27.8 | 3.4 | --- | --- | --- |
| 283 | 8.9 | 3.4 | 2.6 | --- | --- | --- |
| 284 | 8.2 | 29.8 | 3.8 | --- | --- | --- |
| 285 | 11.3 | 19.5 | 2.6 | --- | --- | --- |
| 286 | 7.8 | 18.7 | 2.1 | --- | --- | --- |
| 287 | 6.0 | 12.6 | 1.4 | --- | --- | --- |
| 288 | 5.7 | 5.2 | 3.4 | --- | --- | --- |
| 289 | 3.3 | 5.1 | 3.6 | --- | --- | --- |
| 290 | 6.9 | 56.0 | 9.4 | --- | --- | --- |
| 291 | 6.4 | 10.8 | 3.0 | --- | --- | --- |
| 292 | 4.5 | 4.8 | 6.6 | --- | --- | --- |
| 293 | 2.5 | 9.1 | 1.4 | --- | --- | --- |
| 294 | 7.5 | 19.4 | 6.8 | --- | --- | --- |
| 295 | 15.3 | 29.0 | 2.8 | --- | --- | --- |
| 296 | 5.8 | 20.4 | 4.5 | --- | --- | --- |
| 297 | 2.2 | 19.0 | 2.4 | --- | --- | --- |
| 298 | 3.9 | 40.6 | 6.4 | --- | --- | --- |
| 299 | 5.5 | 26.1 | 10.4 | --- | --- | --- |
| 300 | 8.5 | 45.0 | 9.5 | --- | --- | --- |
| 301 | 13.7 | 15.9 | 1.1 | --- | --- | --- |
| 302 | 6.0 | 0 | 0.2 | --- | --- | --- |
| 303 | 12.8 | 0 | 0.8 | --- | --- | --- |
| 304 | 3.7 | 0 | 0.9 | --- | --- | --- |
| 305 | 15.2 | 1.8 | 1.0 | --- | --- | --- |
| 306 | 0 | 9.2 | 1.4 | --- | --- | --- |
| 307 | 1.3 | 14.6 | 3.7 | --- | --- | --- |
| 308 | 0.4 | 25.4 | 2.5 | --- | --- | --- |
| 309 | 1.5 | 32.1 | 6.2 | --- | --- | --- |
| 310 | 6.0 | 3.7 | 1.8 | --- | --- | --- |
| 311 | 5.5 | 8.6 | 2.7 | --- | --- | --- |
| 312 | 0 | 3.7 | 0.6 | --- | --- | --- |
| 313 | 0 | 4.3 | 0 | --- | --- | --- |
| 314 | 1.9 | 3.7 | 1.4 | --- | --- | --- |
| 315 | 0 | 2.2 | 0.3 | --- | --- | --- |
| 316 | 4.3 | 13.3 | 2.0 | --- | --- | --- |
| 317 | 1.3 | 1.2 | 1.4 | --- | --- | --- |
| 318 | 6.4 | 13.9 | 1.5 | --- | --- | --- |
| 319 | 5.5 | 29.9 | 1.7 | --- | --- | --- |
| 320 | 0 | 0 | 1.3 | --- | --- | --- |
| 321 | 10.8 | 24.0 | 2.6 | --- | --- | --- |
| 322 | 2.5 | 26.3 | 2.4 | --- | --- | --- |
| 323 | 10.2 | 31.0 | 2.7 | --- | --- | --- |
| 324 | 5.2 | 27.9 | 3.2 | --- | --- | --- |
| 325 | 7.4 | 24.0 | 2.7 | --- | --- | --- |
| 326 | 3.9 | 11.4 | 2.0 | --- | --- | --- |
| 327 | 5.2 | 35.8 | 1.3 | --- | --- | --- |
| 328 | 4.7 | 32.1 | 5.5 | --- | --- | --- |
| 329 | 2.8 | 28.9 | 0.5 | --- | --- | --- |
| 330 | 3.7 | 25.3 | 1.9 | --- | --- | --- |
| 331 | 4.0 | 43.4 | 2.9 | --- | --- | --- |
| 332 | 6.9 | 7.2 | 1.7 | --- | --- | --- |
| 333 | 0 | 6.9 | 2.2 | --- | --- | --- |
| 334 | 0.9 | 18.6 | 3.1 | --- | --- | --- |
| 335 | 2.4 | 19.3 | 6.1 | --- | --- | --- |
| 336 | 14.8 | 33.4 | 2.2 | --- | --- | --- |
| 337 | 1.3 | 25.0 | 4.3 | --- | --- | --- |
| 338 | 0.3 | 31.7 | 2.2 | --- | --- | --- |
| 339 | 2.2 | 46.0 | 7.0 | --- | --- | --- |
| 340 | 2.4 | 38.6 | 3.2 | --- | --- | --- |
| 341 | 8.9 | 16.6 | 0.6 | --- | --- | --- |
| 342 | 4.0 | 27.8 | 2.6 | --- | --- | --- |
| 343 | 6.4 | 29.3 | 1.5 | --- | --- | --- |
| 344 | 3.5 | 38.8 | 3.8 | --- | --- | --- |
| 345 | 2.6 | 30.8 | 3.0 | --- | --- | --- |
| 346 | 0 | 21.5 | 0 | --- | --- | --- |
| 347 | 2.4 | 34.4 | 3.0 | --- | --- | --- |
| 348 | 1.7 | 21.4 | 0.9 | --- | --- | --- |
| 349 | 3.2 | 33.1 | 1.0 | --- | --- | --- |
| 350 | 6.0 | 39.5 | 5.9 | --- | --- | --- |
| 351 | 13.3 | 45.0 | 3.2 | --- | --- | --- |
| 352 | 1.5 | 23.2 | 2.1 | --- | --- | --- |
| 353 | 3.0 | 30.9 | 2.5 | --- | --- | --- |
| 354 | 6.4 | 33.5 | 5.1 | --- | --- | --- |
| 355 | 1.9 | 30.9 | 2.4 | --- | --- | --- |
| 356 | 3.1 | 42.7 | 8.5 | --- | --- | --- |
| 357 | 3.0 | 37.6 | 2.7 | --- | --- | --- |
| 358 | 4.5 | 23.2 | 3.4 | --- | --- | --- |
| 359 | 5.0 | 25.8 | 3.5 | --- | --- | --- |
| 360 | 3.1 | 24.1 | 1.4 | --- | --- | --- |
| 361 | 13.6 | 25.1 | 5.6 | --- | --- | --- |
| 362 | 5.6 | 4.7 | 0 | --- | --- | --- |
| 363 | 0.6 | 16.3 | 4.1 | --- | --- | --- |
| 364 | 1.8 | 23.3 | 0 | --- | --- | --- |
| 365 | 10.6 | 0.6 | 0.6 | --- | --- | --- |
| 366 | 5.0 | 15.1 | 3.3 | --- | --- | --- |
| 367 | 5.3 | 4.0 | 0 | --- | --- | --- |
| 368 | 3.0 | 24.5 | 2.7 | --- | --- | --- |
| 369 | 3.3 | 17.0 | 3.2 | --- | --- | --- |
| 370 | 8.1 | 10.2 | 2.6 | --- | --- | --- |
| 371 | 0.8 | 44.7 | 4.6 | --- | --- | --- |
| 372 | 16.5 | 29.6 | 10.8 | --- | --- | --- |
| 373 | 2.3 | 34.7 | 12.6 | --- | --- | --- |
| 374 | 5.1 | 45.4 | 13.4 | --- | --- | --- |
| 375 | 6.0 | 28.8 | 3.9 | --- | --- | --- |
| 376 | 7.1 | 13.7 | 2.7 | --- | --- | --- |
| 377 | 2.3 | 29.1 | 4.6 | --- | --- | --- |
| 378 | 12.7 | 45.9 | 11.7 | --- | --- | --- |
| 379 | 2.7 | 12.1 | 1.0 | --- | --- | --- |
| 380 | 1.1 | 25.9 | 1.0 | --- | --- | --- |
| 381 | 2.9 | 19.3 | 0.7 | --- | --- | --- |
| 382 | 0.1 | 0 | 0 | --- | --- | --- |
| 383 | 6.4 | 23.2 | 0.8 | --- | --- | --- |
| 384 | 2.8 | 0 | 0.2 | --- | --- | --- |
| 385 | 1.8 | 23.9 | 1.2 | --- | --- | --- |
| 386 | 2.7 | 11.5 | 2.2 | --- | --- | --- |
| 387 | 0 | 21.6 | 4.0 | --- | --- | --- |
| 388 | 2.3 | 45.5 | 2.5 | --- | --- | --- |
| 389 | 0 | 29.3 | 2.7 | --- | --- | --- |
| 390 | 0 | 25.4 | 1.1 | --- | --- | --- |
| 391 | 0.2 | 18.1 | 3.5 | --- | --- | --- |
| 392 | 8.1 | 32.6 | 3.7 | --- | --- | --- |
| 393 | 2.1 | 23.8 | 3.4 | --- | --- | --- |
| 394 | 3.0 | 16.5 | 3.8 | --- | --- | --- |
| 395 | 6.6 | 32.3 | 9.5 | --- | --- | --- |
| 396 | 0.9 | 20.7 | 4.9 | --- | --- | --- |
| 397 | 6.7 | 24.1 | 4.1 | --- | --- | --- |
| 398 | 12.4 | 27.7 | 1.8 | --- | --- | --- |
| 399 | 1.8 | 10.0 | 1.2 | --- | --- | --- |
| 400 | 2.1 | 25.7 | 1.0 | --- | --- | --- |
| 401 | 1.4 | 31.4 | 2.7 | --- | --- | --- |
| 402 | 8.2 | 20.5 | 0.4 | --- | --- | --- |
| 403 | 0 | 9.6 | 0.3 | --- | --- | --- |

**Table S1**. Cerebrospinal fluid iron biomarker values for CHARTER study participants at baseline and 6-months.

*Units of measurement:* (CSF) iron, µg/dL; transferrin, µg/mL; ferritin, ng/mL
