# Supplementary material for: Cerebrospinal fluid (CSF) biomarkers of iron status are associated with CSF viral load, antiretroviral therapy, and demographic factors in HIV-infected adults
Source: Fluids Barriers CNS. 2017 Apr 21;14:11. doi: 10.1186/s12987-017-0058-1 (PMC5399327; doi:10.1186/s12987-017-0058-1)
Supplement: Supplementary file 3 — Additional file 3. Visit-matched (6-month) CSF and serum iron biomarker values for 11 CHARTER study participants. Units of measurement: (CSF) iron, µg/dL; transferrin, µg/mL; ferritin, ng/mL; (Serum) iron, µg/dL; transferrin, mg/dL; ferritin, ng/mL. [file 12987_2017_58_MOESM3_ESM.docx]

| **Subject** | **Biomarker** | **CSF** | **Serum** | **Serum:CSF Ratio** |
| --- | --- | --- | --- | --- |
| (1) | Iron | 3.75 | 73.8 | 20 |
|  | Transferrin | 58.67 | 2750 | 47 |
|  | Ferritin | 1.82 | 95.9 | 53 |
| (2) | Iron | 2.88 | 106.5 | 37 |
|  | Transferrin | 16.80 | 3030 | 180 |
|  | Ferritin | 1.81 | 62.9 | 35 |
| (3) | Iron | 4.75 | 85.8 | 18 |
|  | Transferrin | 14.29 | 3140 | 220 |
|  | Ferritin | 7.22 | 118.6 | 16 |
| (4) | Iron | 3.5 | 139.7 | 40 |
|  | Transferrin | 34.94 | 2680 | 77 |
|  | Ferritin | 5.23 | 376.3 | 72 |
| (5) | Iron | 3.63 | 153.2 | 42 |
|  | Transferrin | 0 | 3150 | -- |
|  | Ferritin | 1.17 | 431.2 | 368 |
| (6) | Iron | 1.87 | 242.2 | 129 |
|  | Transferrin | 10.85 | 3110 | 287 |
|  | Ferritin | 1.76 | 264.8 | 150 |
| (7) | Iron | 1.25 | 183.5 | 147 |
|  | Transferrin | 13.84 | 3110 | 225 |
|  | Ferritin | 1.42 | 202.9 | 143 |
| (8) | Iron | 0.25 | 157.9 | 632 |
|  | Transferrin | 6.13 | 3380 | 551 |
|  | Ferritin | 4.70 | 137 | 29 |
| (9) | Iron | 3.88 | 57 | 15 |
|  | Transferrin | 38.41 | 3140 | 82 |
|  | Ferritin | 0.44 | 149.5 | 340 |
| (10) | Iron | 5.50 | 130.5 | 24 |
|  | Transferrin | 51.26 | 4340 | 85 |
|  | Ferritin | 6.82 | 1006 | 148 |
| (11) | Iron | 0 | 99.4 | -- |
|  | Transferrin | 3.15 | 4180 | 1327 |
|  | Ferritin | 8.32 | 240.5 | 29 |

**Table S2**. Visit-matched (6-month) CSF and serum iron biomarker values for 11 CHARTER study participants.

*Units of measurement:* (CSF) iron, µg/dL; transferrin, µg/mL; ferritin, ng/mL; (Serum) iron, µg/dL; transferrin, mg/dL; ferritin, ng/mL
